# The Impact of COVID-19 Infection on Oxygen Homeostasis: A Molecular Perspective

**DOI:** 10.3389/fphys.2021.711976

**Published:** 2021-09-27

**Authors:** Abdu I. Alayash

**Affiliations:** Division of Blood and Devices (DBCD), United States Food and Drug Administration, Silver Spring, MD, United States

**Keywords:** COVID-19, hemoglobin, oxygen transport, hypoxia inducible factor, mitochondrial function, hypoxia

## Abstract

The novel coronavirus (2019-nCoV/SARS-CoV-2) causes respiratory symptoms including a substantial pulmonary dysfunction with worsening arterial hypoxemia (low blood oxygenation), eventually leading to acute respiratory distress syndrome (ARDS). The impact of the viral infection on blood oxygenation and other elements of oxygen homeostasis, such as oxygen sensing and respiratory mitochondrial mechanisms, are not well understood. As a step toward understanding these mechanisms in the context of COVID-19, recent experiments revealed contradictory data on the impact of COVID-19 infection on red blood cells (RBCs) oxygenation parameters. However, structural protein damage and membrane lipid remodeling in RBCs from COVID-19 patients that may impact RBC function have been reported. Moreover, COVID-19 infection could potentially disrupt one, if not all, of the other major pathways of homeostasis. Understanding the nature of the crosstalk among normal homeostatic pathways; oxygen carrying, oxygen sensing (i.e., hypoxia inducible factor, HIF) proteins, and the mitochondrial respiratory machinery may provide a target for therapeutic interventions.

## COVID-19 Infection and Oxygen Homeostasis

The coronavirus, known as Severe Acute Respiratory Syndrome Coronavirus 2 (SARS-CoV-2), is responsible for a multi-systemic disease, called COVID-19. COVID-19 presents with a wide spectrum of clinical signs and symptoms, varying from asymptomatic infection to acute respiratory distress syndrome (ARDS), multifunctional organ dysfunction, and death. Under normal physiological settings the maintenance of cellular levels of oxygen is critical because either insufficient or excess oxygen leads to increased levels of reactive oxygen species (ROS), and therefore, both the delivery and the consumption of oxygen are precisely regulated by many different molecular mechanisms as part of overall oxygen homeostasis. Here, we review the complex and often contradictory literature on the role of blood oxygenation and its impact on other elements of oxygen homeostasis under COVID-19 conditions with a special focus on the delicate balance between oxygen transport/delivery, oxygen sensing (how cells sense oxygen), and finally oxygen consumption (how oxygen is utilized for energy).

## Oxygen Transport Under Normal and COVID-19 Infectious Conditions

### Oxygen Transport Under Normal Physiology

**Figure 1:**
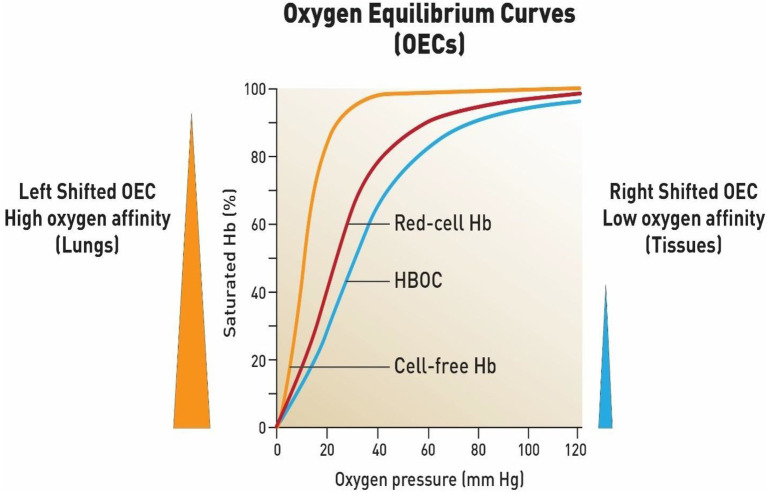
Oxygen equilibrium curves of free hemoglobin, fresh red blood cells and chemically modified hemoglobin-based oxygen carrier (HBOC). First curve on the left represents a typical OEC of an isolated purified Hb (HbA0) (orange line) (P50 = 8- 12 mmHg) (high oxygen affinity). Middle curve is constructed form fresh red blood cells (red line) (P50 = 29-30 mmHg). When free Hb is crosslinked chemically or genetically it produces a right shifted OEC (P50 = 30 to 32 mmHg)) (blue line) which is designed to deliver more oxygen to tissues as a blood substitute. These curves were constructed based on data obtained in the author’s laboratory using a Hemox analyzer under standard experimental conditions (Alayash, 2014).

Hemoglobin (Hb) is the primary oxygen transporting molecule; some 200–300 million molecules are packed inside circulating red blood cells (RBCs). The binding of oxygen (four oxygen per four hemes) process occurs at the lungs. Binding of the first oxygen (oxyHb; bright red) causes a gradual increase in oxygen-binding affinity until all binding sites on the Hb molecule are filled in a cooperative manner. As a result, the oxygen-dissociation curve of Hb (ODC; also called the oxygen equilibrium curve, OEC) is sigmoidal or S-shaped, as opposed to the normal hyperbolic curve associated with non-cooperative binding (e.g., myoglobin; Dickerson and Gies, 1993; Figure 1).

Carried by arteries, RBCs loaded with oxygen are transported to tissues, where several allosteric modifiers, 2,3-diphosphoglycerate (2,3-DPG), pH (H^+^), chlorides (Cl^−^), and CO_2_, collectively force Hb to unload its oxygen at the respiring tissues. Active metabolism within the respiring tissues generates a considerable amount of CO_2_ that must be eliminated to avoid acidosis. Hb returns to the lungs as deoxyHb; it directly carries CO_2_ (20%) back on a single amino acid, Val 1 of the β chain of Hb as a carboamino group. Hb indirectly helps in the removal of dissolved CO_2_ (80%) as it is being converted from its dissolved acid form and exhaled through the lungs. Carbonic anhydrase, an enzyme found in RBCs catalyzes the reaction between carbonic acid, CO_2_, and water (Dickerson and Gies, 1993; Alayash, 2004; Balcerek et al., 2020).

Typically, OECs are generated from a plot of the proportion of Hb in its saturated (oxygen-laden) form on the vertical axis against the prevailing oxygen tension on the horizontal axis (Figure 1). Several important parameters can be derived from these OECs by applying appropriate calculations from the Hill equations. The P50 value when Hb is half saturated and Hill coefficients (n50) as well as cooperativity (*n*50) can be derived from these formula. Another important parameter is whether the OECs are left shifted (high oxygen affinity with small P50) or right shifted (low oxygen affinity with a large P50; Alayash, 2004).

### Oxygen Transport Under COVID-19 Conditions

Blood oxygen levels can be assessed by the following oxygenation parameters; SaO_2_, SpO_2_, and PaO_2_. SaO_2_ is the oxygen saturation of arterial blood, while SpO_2_ is the arterial oxygen pressure as detected by a pulse oximeter. PaO_2_ represents the arterial blood gas (oxygen tension). The amount of oxygen bound to Hb will increase as the partial pressure of oxygen increases. SaO_2_, in arterial blood is normally >95%, whereas at the venous outflow, the venous oxygen saturation (SVO_2_; Hb returning to heart without oxygen) is about 65–75%. Normal levels of PaO_2_ and PaCO_2_ are reported in the range of 75–100 and 38–42mm Hg, respectively (Bickler et al., 2017).

In a recent study on several COVID-19 patients (three patients) ranging in age from 58 to 74 years, the following oxygenation parameters were reported; SaO_2_ ranged between 69 and 75%, SVO_2_ ranged between 68 and 76, and SPO_2_ it was reported to be between 68 and 76%. Partial pressure of oxygen and CO_2_ were reported to be between 36 and 45 and 34–40mm Hb, respectively (Tobin et al., 2020).

Accordingly, novel treatments have been considered in order to improve Hb-based delivery of oxygen molecules to peripheral tissues in COVID-19 patients. This includes hyperbaric oxygen therapy that significantly increases oxygen levels in the blood independently of Hb levels and improves tissue oxygenation (Pan et al., 2020). Packed RBCs for transfusion or injected erythropoiesis-stimulating agents that can significantly raise blood Hb levels by increasing the numbers of RBCs and improving tissue oxygenation have been suggested (Geier and Geier, 2020).

Manufacturers of Hb-based blood substitutes have also promoted the use of oxygen therapeutics during the current COVID-19 pandemic (Weiskopf et al., 2020; Lupon et al., 2021). These oxygen therapeutics have a long shelf-life and can be stored at room temperatures for long durations.

### Contested Effects of Severe COVID-19 Infection on Oxygen Transport and Red Blood Cell Integrity

The question that remains unanswered is whether we know the full impact of COVID-19 infection on the blood oxygenation parameters in the first place. One of the early studies (Daniel et al., 2020) on Hb oxygen affinity measurements were recorded in 14 patients with COVID-19 and reported similar blood oxygenation to the values obtained from 11 control participants. The oxygen affinity was measured *in vitro* using the automated Hemox Analyzer under standard conditions [pH (7.4) and room temperature]. The (P50) values were obtained directly from the analyzer, without adjustments for physiological changes in CO_2_ or pH *in vivo*, which could be important in COVID-19.

Clinical data, specifically blood oxygen parameters from 21 critically ill COVID-19 patients and 21 non-COVID-19 ARDS (patient controls), were used to generate Hb-ODCs from direct measurements of venous blood gases. The ODC curve generated from the COVID-19 cohort matched the normal sigmoidal ODC well from normal subjects. This comparison of ODC is not altered in patients with COVID-19 admitted to the ICU. Corresponding lab values of Hb, total bilirubin, ferritin, iron, and LDH were similar between ICU patients with COVID-19 and those with ARDS without COVID-19 (DeMartino et al., 2020).

To assess alterations in the *in vivo* Hb oxygen affinity, a retrospective, observational analysis of all arterial and venous blood gases obtained from all intubated and ventilated patients (*n*=43) with severe COVID-19 in an intensive care unit was also reported (Vogel et al., 2020). In this study, the P50 values were calculated using the Hill equation after correcting for pH, temperature, and base excess derived from a Roche Blood gas analyzer and compared to the normal value (for pH 7.4, 37.0°C and pCO_2_ 40mmHg). Using a reported normal P50 of 26.7mm Hg, distribution of P50 values calculated using Hill equation from measured SpO_2_ and SaO_2_ showed a left shift in OECs (P50 values ranged between 14.7 and 25.7mmHg) for the bulk of the patients and smaller number of patients exhibited a right shift of oxy Hb affinity from the standard P50 value (Vogel et al., 2020).

In a more recent retrospective observational study, blood samples from 100 subjects (COVID and non-COVID) from each group were analyzed. The time-course of P50 between days 1 and 18, showed no significant differences among the groups. Median P50 at baseline was 26mmHg (25.2–26.8) vs. 25.9mmHg (24–27.3), respectively (Gille et al., 2021).

Another unresolved aspect of this disease is the impact COVID-19 infection on the health and integrity of circulating RBCs in these patients. Early modeling studies by Liu and Li (2020), predicted the spike S1 protein can interact with Hb to reduce both oxygen affinity and total Hb content, but the work was subsequently questioned due to a lack of experimental support (Read, 2020).

Using state-of-the-art metabolomics, proteomics, and lipidomics approaches (Thomas et al., 2020), the impact of COVID-19 was investigated from RBCs obtained from 23 healthy subjects and 29 COVID-19 patients. RBCs from COVID-19 patients had increased levels of glycolytic intermediates, accompanied by oxidation and fragmentation of ankyrin, spectrin beta, and the N-terminal cytosolic domain of band 3 (AE1). These increases in RBC glycolytic proteins were reported to be consistent with a theoretically improved capacity of Hb to off-load oxygen modulation by high-energy phosphate compounds, perhaps to counteract COVID-19-induced hypoxia. It is also known that the N- terminus of AE1 stabilizes deoxyHb and finely tunes oxygen off-loading and metabolic rewiring toward the hexose monophosphate shunt, in RBCs from COVID-19 patients (Thomas et al., 2020).

The basic understanding of cellular oxygen availability under COVID-19 conditions is not fully understood and is rather controversial. For oxygen to reach cells and ultimately the mitochondria for oxidative metabolism it requires normal blood oxygen carrying capacity as determined by Hb level, and its ability to release oxygen to the tissue (Kulow and Fähling, 2021). It appears as indicated, by only a few investigations so far, that both oxygen carrying capacity as well RBC integrity, are affected under COVID-19 conditions.

## Oxygen Sensing Mechanisms and the Hypoxia Inducible Factor-1α

**Figure 2:**
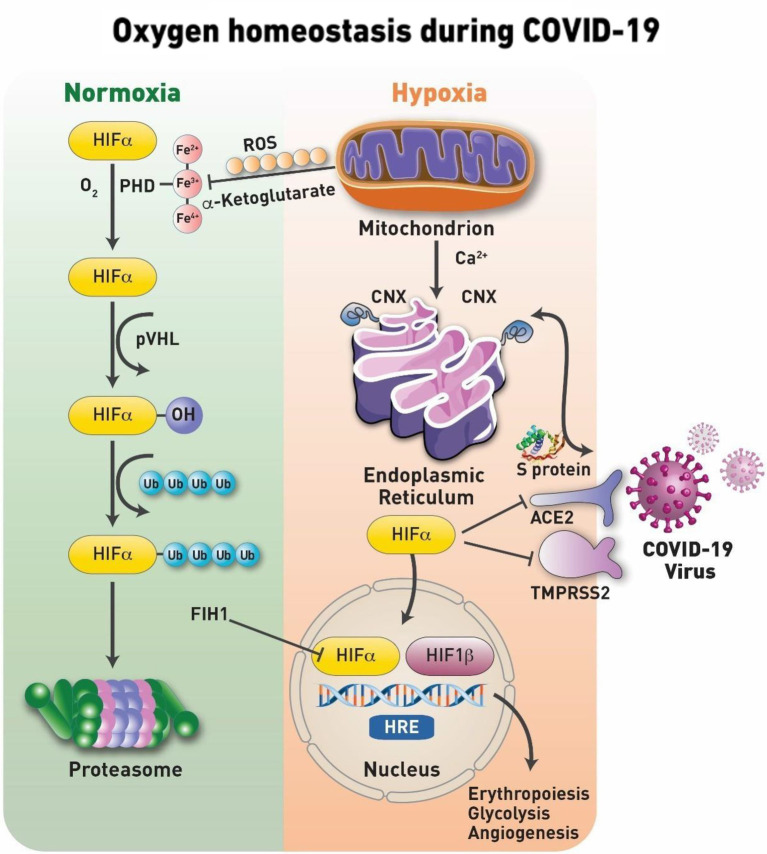
Oxygen homeostasis during COVID-19. Under normal physiological oxygen levels (normoxia), the first step in the hypoxia-inducible factor (HIF-1α) degradation is an oxygen-dependent interaction with the von Hippel– Lindau (VHL) tumor suppressor protein (pVHL) complex. This requires hydroxylation of two HIF-1α proline residues by a family of α-ketoglutarate-dependent dioxygenases, prolyl hydroxylases (PHDs), which requires oxygen, iron, ascorbate and α-ketoglutarate to function. The catalytic cycle in the PHD involves the transition of its iron from Fe2+ to Fe3+ and most active Fe4+ states. Following hydroxylation, HIF-1α subunits are polyubiquitylated by pVHL and targeted for proteasomal degradation. Under conditions of oxygen deprivation, mitochondria increase their production of reactive oxygen species (ROS). Serving as signaling molecules, these ROS inhibit the hydroxylation of HIF-1α and, thus, preventing its proteasomal degradation. The ROS are known to target the iron transition required for the protein hydroxylation and/or specific cysteine residues for the irreversible oxidation of this amino acid that results in the deactivation of the protein. Stabilized HIF-1α is able to translocate to the nucleus where it forms a heterodimeric complex with HIF-1β/ARNT. Factor inhibiting HIF-1 (FIH-1), is a novel protein that interacts with HIF-1α and mediates repression of HIF-1α transcriptional activity. The transcription factor complex activates the expression of several genes, such as Epo and Vegf, to increase oxygen delivery and many others including pyruvate dehydrogenase 1 (PDK1) to reduce oxygen consumption. These factors secure oxygenation to the tissues. At the onset of infection and activation of HIF-1α which is shown to suppress the angiotensin-converting enzyme 2 (ACE2) receptor and transmembrane protease serine 2 (TMPRSS2) and upregulate disintegrin and metalloproteinase domain-containing protein 17 (ADAM17). In addition, the protein targets of HIF-1α are involved with the activation of pro-inﬂammatory cytokine expression and the subsequent inﬂammatory process. In addition, coronaviruses, as well as other viruses, use the host’s chaperone system for the folding of their proteins and for viral assembly, translocation to the nucleus, and other vital steps. Calnexin (CNX) is one of those chaperone proteins of the endoplasmic reticulum (ER) system that ensures the proper folding of viral glycoproteins. In addition, CNX is involved in trafficking of calcium (Ca^2+^) during ER- mitochondria Ca^2+^ flux, maintaining the necessary redox balance within the mitochondria.

Hypoxia inducible factor (HIF) together with its iron containing enzyme, prolyl hydroxylase (PHD) has been identified as key elements in tissue sensing mechanisms. Under normal oxygen tension (normoxia), the transcriptional activity of HIF-1α is halted by a process of hydroxylation (at Pro564 and/or Pro402), which involves the non-heme iron α-ketoglutarate dependent dioxygenases, PHD (Semenza, 2007). The iron center (Fe^2+^) of the PHD enzyme undergoes redox transition to ferric (Fe^3+^) and ferryl (Fe^4+^), which appears to be the main regulator of HIF-1α protein stability and control (Chowdhury et al., 2009). This process is followed by ubiquitination of some lysine residues prior to the final degradation by proteasome. During the initial stages of hypoxia, HIF, which contains the two subunits, an α-subunit that quickly degrades in the presence of oxygen (its half-life is less than 5min in 21% oxygen) and a more stable β is translocated to the nucleus, where it binds to hypoxia response element a process that results in activation of a number of target genes, which collectively function to correct oxygen deficits. This specifically includes a group of genes participating in glucose metabolism, control of intracellular pH, angiogenesis, erythropoiesis, and mitogenesis (Semenza, 2007; Figure 2).

One of the early experiments demonstrating a link between blood oxygen levels and HIF involved exchanging 50% of rat blood with non-oxygen-carrying pentastarch. In this model, hypoxia led to increased expression of HIF-1*α*, and several other target genes such as endothelial nitric oxide synthase (eNOS) and vascular endothelial growth factor (VEGF) in the cerebral cortex of these mildly anemic rats (McLaren et al., 2007). To test whether an oxygen carrier cross talks with HIF under hypoxic conditions in an *in vivo* setting, a class of therapeutics designed to correct oxygen deficit under conditions of anemia and traumatic blood loss were used. A correlation between the oxygenation/oxidation states of the cell-free Hb and renal HIF-1*α*-binding activity in a rat and guinea- pig model of 50% blood replacement was observed (Buehler et al., 2007). Using a more severe model of hemodilution designed specifically to induce hypoxia (80% ET; exchange transfusion), a polymerized oxygen carrying Hb suppressed HIF expression in kidney tissues for approximately 5h post infusion as compared to non-oxygen carrying hetastraches. There was a corresponding depression in EPO gene expression as well EPO serum levels in these animals followed by gradual rebound in EPO as Hb is cleared (half time of ~16h) and/or due to complete oxidation of the protein (Manalo et al., 2008). These studies collectively demonstrated in an *in vivo* setting the delicate balance that existed between oxygen content of blood and oxygen sensing mechanisms in tissues.

### HIF and Oxygen Sensing Pathways Under COVID-19 Infection Conditions

Globally, hypoxia impacts organs at the cellular and subcellular levels. Hypoxic responses and hypoxia-mediated elements have been reported in severe cases of COVID-19, which may progress to ARDS, ultimately leading to end organ dysfunction and failure (Serebrovska et al., 2020). At the cellular level, once cells are infected with SARS-CoVID, accumulation of HIF-1α may occur due to increased expression as well as inhibited proteasome degradation (Vassilaki and Frakolaki, 2017). In addition, a possible secondary bacterial infection during the later phase of COVID-19 may result in the stabilization of HIF-1α in macrophages *via* the activation of toll-like receptor 4 (TLR4) and decrease in prolyl hydroxylase mRNA in severe inflammatory vascular disease (Peyssonnaux et al., 2007). Subsequent local hypoxia events may also occur when leukocytes are activated in response to secreted interferon as well as to accumulation of pathogen associated molecular patterns (PAMPs) and damage associated molecular patterns (DAMPs) as part of the normal innate immune responses.

The entry of the SARS-CoV-2 virus inside cells is facilitated by angiotensin converting enzymes (ACE) found in the lungs, kidneys, heart, and arteries (Gheblawi et al., 2020). Under hypoxic conditions ACE-1, is upregulated by the (HIF-1); meanwhile, the expression of ACE-2 is markedly decreased (Figure 2). It has been suggested that increased levels of ACE-2 were positively associated with COVID-19 infection (Koch et al., 2020).

However, there appears to be a disconnect according to recent reports between hypoxemia (low arterial oxygen saturation) in those patients who generally experience only mild respiratory distress or difficulty in breathing (dyspnea; Tobin et al., 2020). Several other seemingly contradictory observations have also been made. For example, severe hypoxemia in COVID-19 patients does not necessarily cause tissue hypoxia. This may be due to a lack of understanding in the pathophysiology of what has been described as “happy hypoxia” (Rahman et al., 2021). However, a variety of factors have been reported to play a possible role in this phenomenon that includes, a left shift in Hb’s OECs, low levels of CO_2_, decreased pH, and potentially changes in 2,3-DPG levels (Dhont et al., 2020).

## Mitochondrial Respiration Under Normal and COVID-19 Infection Conditions

Mitochondria are essential cellular organelles that play important roles in regulating cellular energy, metabolism, survival, and proliferation (Nakhle et al., 2020). Their main energy currency is adenosine triphosphate (ATP) generated by the electron transport chain (ETC). ETC is the metabolic bottle neck, where breakdown metabolites resulting from food degradation pathways (i.e., glycolysis, amino acid, and lipid degradation) followed by the tricarboxylic acid cycle (TCA) are processed. These metabolites are then transformed into protons (electrons) tunneled through the ETC complexes (I, II, III, and IV) that are largely bound to the inner membrane of the mitochondria. As electrons shuttled through the ETC chain, ATP is produced as a result of a well-orchestrated oxidation phosphorylation (OXPHOS) process. Mitochondria also provide intracellular signaling messages through ROS production (Martínez-Reyes and Chandel, 2020). As expected with such a central cellular role, mitochondrial dysfunctions have been linked to many different diseases. Quantifying bioenergetic health index (BHI) has become an important tool in patient populations as laboratory methods have been developed to begin an integrated approach in cells isolated from human blood to establish a quantitative assay of mitochondrial function that will have the power to predict disease progression and response to treatment (Chacko et al., 2014).

Mitochondrial dysfunction and subsequent pathogenesis due to multi-organ failure has been linked to COVID-19 infection and is under active investigation. Recent reports suggest that the SARS-CoV-2 virus proteins such as the spike protein interacts with host cell mitochondrial proteins leading to loss of membrane integrity and dysfunction in the bioenergetics of the mitochondria. These mitochondrial proteins may also serve as damage-associated molecular pattern (DAMP) molecules, which activate innate immunity (Shenoy, 2020).

A recent paper suggested that COVD infection evade host immunity by “hijacking” mitochondrial pathways. Specifically, this effect is manifested into critical areas, such as the lungs where mitochondrial dysfunction due to SARS-CoV2 infection possibly contributes to pulmonary tissue damage, deterioration of pulmonary function, and airway hypoxia. Impaired mitochondria in the carotid bodies may also worsen hypoxemia due to impaired oxygen sensing and result in a compromised chemoreflex (Burtscher et al., 2020). In a recent study, functional mitochondrial changes in live peripheral blood mononuclear cells (PBMCs) from patients with COVID-19 were investigated. The investigators demonstrated mitochondrial dysfunction, metabolic alterations with an increase in glycolysis, and high levels of mitokine in PBMCs from patients with COVID-19. These data suggest that patients with COVID-19 have a compromised mitochondrial function and an energy deficit that is compensated by a metabolic switch to glycolysis. This metabolic manipulation by SARS-CoV-2 triggers an enhanced inflammatory response that contributes to the severity of COVID-19 symptoms (Ajaz et al., 2021).

Using a conventional Seahorse XF analyzer, two important parameters were measured in the presence of specific mitochondrial activators and inhibitors; oxygen consumption rate (OCR), a measurement of mitochondrial respiration, and extracellular acidification rate (ECAR), which correlates to the number of protons released from the cell (due to contribution from glycolysis and the Krebs cycle; Ajaz et al., 2021). The authors reported a lack of significant differences in basal and stressed OCR among COVID-19-positive individuals’ patients. Stressed ECAR was also higher as compared with controls. This high basal and stress ECAR suggest that the PBMCs of patients with COVID-19 depend on glycolysis for energy (Ajaz et al., 2021).

## COVID-19 Infection Disrupts Homeostatic Pathways-A Working Model

Figure 1 represents a working model that combines the major pathways of homeostasis under normal oxygen tension (normoxia) and during oxygen deprivation (hypoxia) and the impact of COVID-19 infection on these pathways. Among the key signaling molecules that bridge the mitochondrial and oxygen sensing pathways are the mitochondrial ROS. ROS are generated as a function of decreasing oxygen tension inhibit HIF-1*a* prolyl hydroxylation and degradation.

Therefore, mitochondrial electron transport and subsequent oxidative phosphorylation are interconnected as signaling mechanisms conveying cellular oxygen availability to oxygen sensing pathways (Maltepe and Saugstad, 2009). Increases in other glycolytic intermediates (e.g., α-ketoglutarate) levels results in HIF stabilization (Lee et al., 2020).

Another key player activated during hypoxia is calnexin (CNX); this membrane–bound endoplasmic reticulum (ER) chaperone protein, ensures the proper folding of glycoproteins destined for the plasma membrane or secretion (Kozlov and Gehring, 2020). The SARS-CoV spike glycoprotein (S protein), a key molecule for viral entry, binds to CNX, conferring infectivity on SARS-CoV (Fukushi et al., 2012). Under cellular stress induced by hypoxia, cells display enhanced and sustained expression of the ER stress-related chaperone proteins CNX (Figure 1). CNX controls mitochondrial positioning and respiration, and it appears to be yet another molecule that can bridge the gap between oxygen sensing and mitochondrial pathways even closer (Gutiérrez et al., 2020).

Toward this end, we have recently begun to investigate the interplay between oxygen transport, oxygen sensing, and mitochondrial metabolism in human pulmonary arterial endothelial cells (HPAEC) by exploring, first the impact of COVID-19 spike protein on these parameters in the presence of Hb and under normoxic and hypoxic cell culture conditions. We found that the spike protein alone induced changes in cells without the entry of the virus and that cell-free Hb did not attenuate the effects of the SARS-CoV-2 S1 spike protein *in vitro* (Jana et al., 2021). We also begun to investigate the three elements of homeostasis (oxygen transport, oxygen sensing, and mitochondrial pathways) in lungs from hamster model of COVID-19 infections (Selvaraj et al., 2021) to verify the interconnectivity of these pathways in living biological systems.

COVID-19 infection clearly impacts oxygen sensing elements as seen in changes with HIF-target genes as well as mitochondrial respiration in response to low oxygen levels in circulation. Once these pathways and their relationships are established in clinical settings therapeutically targeting each individual pathway or as a group would be possible. Manipulation of RBC’s oxygen affinity in circulation can be targeted by a number of allosteric modifiers that can shift OECs to either to the left or to the right of normal OECs (Woyke et al., 2021). Stabilizing HIF under normoxic conditions are also feasible by targeting the heme iron of the enzyme PHD or some of the small molecule drugs that are currently used in cancer therapy (Bao et al., 2021).

Finally, mitochondrial or glycolytic intervention are also feasible by targeting a site or sites in the mitochondrial respiratory chain using small molecule drugs (Andreux, 2013).

## Data Availability Statement

The original contributions presented in the study are included in the article/supplementary material, further inquiries can be directed to the corresponding author.

## Author’s Note

This article reflects the views of the author and should not be construed to represent FDAs views or policies.

## Author Contributions

The author confirms being the sole contributor of this work and has approved it for publication.

## Conflict of Interest

The author declares that the research was conducted in the absence of any commercial or financial relationships that could be construed as a potential conflict of interest.

## Publisher’s Note

All claims expressed in this article are solely those of the authors and do not necessarily represent those of their affiliated organizations, or those of the publisher, the editors and the reviewers. Any product that may be evaluated in this article, or claim that may be made by its manufacturer, is not guaranteed or endorsed by the publisher.
